# Transcriptome analysis of leaf and root of rice seedling to acute dehydration

**DOI:** 10.1186/1939-8433-6-38

**Published:** 2013-12-16

**Authors:** Pham-Thi Minh-Thu, Duk-Ju Hwang, Jong-Seong Jeon, Baek Hie Nahm, Yeon-Ki Kim

**Affiliations:** 1 Division of Bioscience and Bioinformatics, Myongji University, Yongin, Kyonggido 449-728, South Korea; 2 Rural Development Administration, National Academy of Agricultural Science, Suwon, Kyonggido 441-707, South Korea; 3 Graduate School of Biotechnology, Kyung Hee University, Yongin, Kyonggido 446-701, South Korea; 4 Genomics Genetics Institute, GreenGene BioTech Inc. Yongin, Yongin, Kyonggido 449-728, South Korea

**Keywords:** Rice, Microarray, Gene ontology, Acute dehydration, ABA, Osmoprotectant, Antioxidant, Photosynthesis

## Abstract

**Background:**

Water deficiency is one of the most serious worldwide problems for agriculture. Recently, it has become more serious and outspread, which urgently requires the production of drought-tolerant plants. Microarray experiments using mRNA from air-dried leaves and roots of rice were performed in an attempt to study genes involved in acute dehydration response.

**Results:**

Set of 10,537 rice genes was significantly up- or down-regulated in leaves or roots under the treatment. Gene Ontology analysis highlighted gene expression during acute dehydration response depending on organ types and the duration of stress. Rice responded by down-regulating many processes which are mainly involved in inhibiting growth and development. On the other hand, phytohormones (ABA, cytokinin, brassinosteroid) and protective molecules were induced to answer to multiple stresses. Leaves induced more genes than roots but those genes were scattered in various processes, most significantly were productions of osmoprotectants and precursors for important pathways in roots. Roots up-regulated fewer genes and focused on inducing antioxidants and enhancing photosynthesis. Myb, zf-C3HC4, and NAM were most strongly affected transcription factors with the dominance of leaf over root.

**Conclusions:**

Leaf and root tissues shared some common gene expression during stress, with the purpose of enhancing protective systems. However, these two tissues appeared to act differently in response to the different level of dehydration they experience. Besides, they can affect each other via the signaling and transportation system.

## Background

Water deficiency is one of the most common environment stresses that negatively influence plant survival, biomass production and crop yield. Most of the world’s agriculture is subject to drought, and the area of land suffering from a water deficiency is increasing. Thus, the need for drought tolerant plants is critical. However, very few crop varieties with improved stress tolerance have been generated by traditional breeding strategies (Agarwal et al. 2006). Recently, several different genetic engineering approaches appear to be more attractive and rapid, but relatively little progress has been made due to the limitation in understanding the drought response mechanism of plants.

Plants respond to abiotic stresses by exhibiting many physiological and developmental changes (Shinozaki and Yamaguchi–Shinozaki 2000, Yu et al. 2013). Some responses may occur very rapidly, within a few seconds, such as changes in the phosphorylation status of proteins. Others changes occur slowly, within minutes and hours, such as changes in gene expression (Bray 1997). For the induction of these stress-inducible genes, plants have at least two major pathways, an abscisic acid (ABA)-dependent and an ABA-independent pathway (Tian et al. 2005). The products of these genes can be classified into two groups: those that directly protect against environmental stresses, and those that regulate gene expression and signal transduction in the stress response. The first group includes proteins that likely function in stress tolerance, such as water channel proteins involved in altering cellular water potential and lipid desaturases for membrane modification. Also, the first group includes enzymes required for the biosynthesis of various osmoprotectants, such as sugars, proline, and betaine. Direct protective proteins such as late embryogenesis abundant (LEA) proteins, osmotin, antifreeze proteins, chaperones, and mRNA-binding proteins are also produced under stress condition. Moreover, thiol proteases, Clp protease, and ubiquitin, which are required for protein turnover, and detoxification proteins such as glutathione S-transferase, soluble epoxide hydrolase, catalase, and ascorbate peroxidase are functional proteins in the first group. The second group contains transcription factors, TFs, (bZIP, MYC, MYB, and DREB), protein kinases, and enzymes involved in phosphoinositide metabolism (Agarwal et al. 2006; Yu et al. 2013). Some stress-tolerant plant phenotypes have been produced by overexpressing stress-inducible genes in transgenic plants, indicating positive functions for those gene products in stress tolerance (Cushman and Bohnert 2000; Bhatnagar–Mathur et al. 2008; Hu et al. 2013; Liu et al. 2013; Yu et al. 2013). However, introducing stress-inducible genes does not always result in a stress tolerant plant because the induction may indicate a highly responsive drought tolerance mechanism or simply a particular sensibility to drought (Price et al. 2002).

Rice (*Oryza sativa*) is an important agronomic crop, also a model monocot plant. The recent completion of the whole genome sequence and the genetic tools available for rice, such as marker maps (Price et al. 2002; International Rice Genome Sequencing Project 2005; Salunkhe et al. 2011), proteomic analysis (Rabello et al. 2008; Choudhary et al. 2009; Degenkolbe et al. 2013), and microarrays (Rabbani et al. 2003; Rensink and Buell 2005; Degenkolbe et al. 2009, Degenkolbe et al. 2013), are advantages to researchers who are studying the drought tolerance of this plant. Microarray technology has been applied to profile gene expression in response to abiotic stresses, such as drought, high salinity, cold, or ABA treatments in several model plants including *Arabidopsis* and rice (Kreps et al. 2002; Seki et al. 2002; Rabbani et al. 2003; Lenka et al. 2011). When microarray technology was initially applied, individual gene expression related to abiotic stress was more or less of interest. Rabbani et al. (2003) analyzed stress-inducible genes in rice using a microarray containing approximately 1700 rice cDNAs. A total of 73 genes were reported as stress-inducible, including 58 novel unreported genes in rice. Among them, 36, 62, 57, and 43 genes were induced by cold, drought, high salinity, and ABA; respectively. The gene expression profile of drought-tolerant and drought-sensitive rice cultivars that were treated for 18 days of drought stress in climate chamber was also surveyed with the 20 K NSF oligonucleotide microarray (Degenkolbe et al. 2009). Localizing all expressed genes on the rice genome map, genes with significant genotype × environment (G × E) interaction co-localized with quantitative trait loci regions for drought tolerance. Microarray analysis was also utilized to profile genome expression changes in rice organs such as shoot, flag leaf, and panicle under drought or high-salinity conditions (Zhou et al. 2007). Patterns of gene expression in response to drought or high-salinity stress showed significant overlap within a particular organ type and unique patterns among different organs.

The application of whole-genome expression microarray usually produces large data sets, which are very difficult to analyze manually. The recent development of bioinformatics and computational biology provides the chance to exploit the database potential by organizing and displaying genes in the context of pre-existing biological knowledge. The Gene Ontology project has developed three structured controlled vocabularies (ontologies) that describe gene products in terms of their associated biological processes, cellular components and molecular functions in a species-independent manner (Ashburner et al. 2000). The project has given biologists excellent opportunities to address complicated gene expression in consistent descriptions of gene products in different databases. Developed on GO category, GoMiner (Ashburner et al. 2000; Zeeberg et al. 2003) is a tool for biological interpretation of ‘omic’ data – including data from gene expression microarrays. It facilitated the analysis of microarray by classifying the genes into biologically coherent categories and assessing these categories. Microarray data can also be accessed by a biochemical aspect: the gene can be classified into biochemical pathway(s) using the AraCyc database for *Arabidopsis* at aracyc_dump_20051021 (http://www.Arabidopsis.org/biocyc). A rice gene is considered to be involved in the pathway if an *Arabidopsis* homolog is part of the pathway.

In this research, an array of 27,448 rice genes was used to elucidate gene expression in air-dried rice seedlings at various periods of time treatment. The analyses show that rice responds to dehydration mainly by down-regulating many biological processes including gene expression and regulation, protein phosphorylation, cellular metabolism. Among strategies to actively adapt to water loss, most significance is inducing protective molecules, which may be differentially regulated based on plant organs.

## Results

### Responses of rice seedling to acute dehydration

The experiment was designed to test genome-wide gene expressions in the leaves and roots of rice seedling through accumulate dehydration. Rice at 14-day-old stage is very fragile and sensitive to abiotic stresses and has been used in many studies (Ferdose et al. 2009; Hua et al. 2009; Gao et al. 2011; Zhou et al. 2011; Hue et al. 2013); hence this stage was selected for experimentation. To get the acute dehydration stress in a short time, we remove the plants from soil and air-dry them. A similar way to conduct the drought treatment was used in previous reports (Dubouzet et al. 2003; Oh et al. 2005; Zhou et al. 2007). Long term in-soil gradual drought treatment could be more natural but complicated by plant’s own development and/or daylight circadian rhythm. In the other hand, removing plants from soil from the beginning helped to collect roots easier.

**Figure 1:**
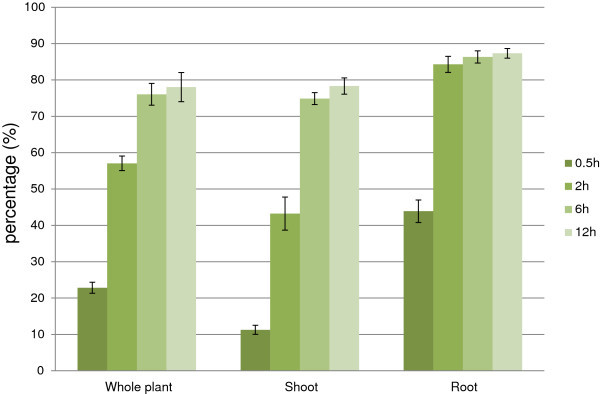
**Fresh weight loss of rice plant during dehydration.** The y axis value is in percentage. Value = mean ± SD (n = 3).

To access the level of water loss during stress treatment, whole plant, shoot and root fresh weights (PFW, SFW and RFW) were measured and FW losses were calculated (Figure 1). After 30 min, about 23% of PFW was lost, the plant was still healthy as there was no symptom of leave rolling or drying with a light level of water loss (SFW loss was about 11%). However, RFW was reduced almost 44% indicating the faster drying out of root. After 2 h of air-dry, with 43% of FW was lost, the leaves were rolled but still maintained the healthy green color. The PFW loss reached 57% because of the strong FW loss of root (84%). After 6 h, leaves were tightly rolled and curled, 1 or 2 leaves still have the healthy color which eventually lost at 12 h. FW loss in root at 6 and 12 h were basically similar to that at 2 h. Based on the Standard Evaluation System for Rice in drought issued by IRRI (Additional file 1: Table S1), the leave appearance at time point 30 min, 2 h, 6 h, and 12 h (Additional file 1: Figure S1) can be classified into scale 0, 3, 7 and 9; respectively. Compare to root, leaf FW loss was slower (Figure 1); thus, water stress was likely to happen earlier in root and the stress signal would travel from root to leaf, as in natural drought condition (Schachtman and Goodger 2008).

In the field, the response of plants to soil water deficits can be divided into three continuous stages (Drought Frontier Project 1999). In stage 1, when water is freely available from the soil, plants are under normal development. Stage 2 starts when water uptake from soil is less than potential transpiration rate of plant; plants maintain the water balance by reducing stomatal conductance to a rate similar to that of uptake from soil. Stage 3 begins when there is no more FTSW, Fraction of Transpirable Soil Water, the stomatal conductance is at minimum level but still excesses the water uptake from soil. When plants are removed from soil and air-dried as in this research, they are likely to enter stage 3 immediately. Because there is no more water uptake through root, fast rate of fresh weight loss is expected. It is a truth that any comparisons between acute dehydration and drought in the field would be relative. However, to examine transcriptome of roots, they must be out of the soil. Some research used the hydroponics system which avoids any possible injury when taking out plants from soil (Kilian et al. 2007; Rabello et al. 2008; Zhou et al. 2011). However, it has its own disadvantage that the nature of plant in hydroponics culture is supposed to be different from that in soil.

To test how the treatment affects gene expression, markers known to be induced, rab21 and dip1, or repressed, rbcS, by water deficit were examined (Mundy and Chua 1988; Reddy et al. 2002; Oh et al. 2005; Ali and Komatsu 2006). As measured by our microarray experiment, rbcS is highly active in leaf and its expression decreased by the duration of dehydration which reached 70% in 12 h of treatment. In contrast, rab21 and dip1 are induced in 2 h and 30 min, respectively and maintained their expression up to 12 h, while ubi is known to be constitutively expressed kept unchanged during the experiment (Additional file 1: Figure S2a). Among the most highly induced genes, there were a putative actin depolymerization factor, ADF, Os03g0820500 and an AWPM-19-like protein, AK102039 (Additional file 1: Figure S2a). The expression level of these 2 genes and 3 markers above was examined by sqRT-PCR to validate the microarray data accuracy (Additional file 1: Figure S2b). ADF, rab21, dip1 were strongly induced in both tissues by dehydration. AWPM was up-regulated in root while rbcS is reduced during treated time in a leaf-specific manner. Thus, basically, our microarray data can represent the expression level of genes.

### Dominance of repression in changes of biological processes and relatively active response of roots compared to leaves during dehydration

The first step to analyze the change of gene expression in response to acute dehydration is to summarize the numbers of 2-fold up/down-regulated genes during the treatment. Among rice genes, 7,308 (26.6%) and 7,104 (25.9%) from leaves and roots; respectively, were 2-fold up- or down-regulated with adjusted p-values less than 0.05. Together, 10,537 genes were either significantly up- or down-regulated during 30 minutes to 12 hours of drought treatment (Additional file 2: Table S2). 1,348 genes (4.9%) were up-regulated in both leaf (3,922) and root (2,147). Similarly, indices of down-regulated genes were 3,336 (12.1%) in both leaf (4,938) and root (5,895). Generally, the number of up-regulated genes was less than that of down-regulated genes in both leaves and roots, indicating the strong negative effect of stress on plant normal growth and this was expected because such a fast water loss as in this research would result in dominance of injury over acclimation (Bray 1997). However, various tissues behaved differently during the stress response. In term of the number of genes, there appeared to be a balance between induction and repression in the leaf, while repression was primarily observed in the root (Additional file 1: Figure S3).

**Figure 2:**
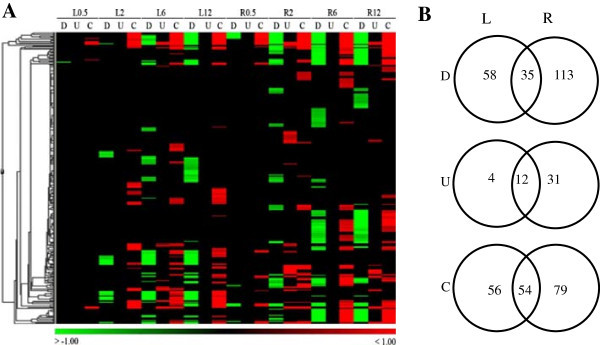
**Enriched biological process in leaves and roots of rice during acute dehydration. (A)** Gene Ontology terms are represented by color lines in Hierachical clustering. Terms that are up-regulated or changed are red, down-regulated are green. **(B)** Total number and overlap GOs between leaves and roots are shown in the Venn diagram. D, U, and C: down, up and change. L/R0.5, 2, 6, 12: leaf/root at 0.5, 2, 6 and 12 h of stress treatment.

**Figure 3:**
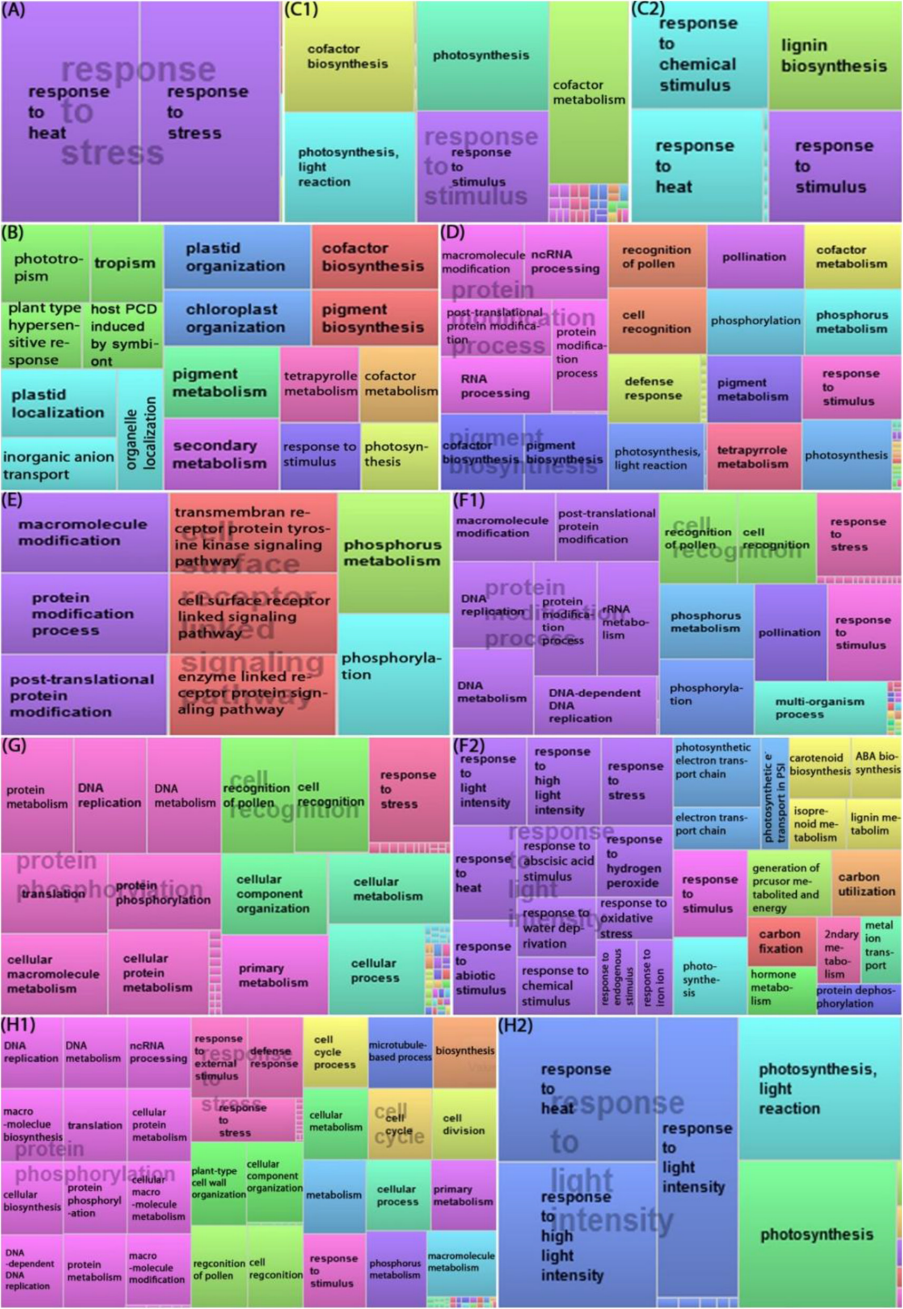
**TreeMap view of GO terms in rice leaf and root.** Each rectangle is a single cluster representative, equal to a GO term. The representatives are joined into “superclusters” of loosely related term, visualized with different color. Size of the rectangles are adjusted by –log10 (p-value). Color and size are not related by time point and mode (i.e. color and size in **A** are different of those in **B**, those in **B** are different in **C1**, ect). **A.** L0.5_C, **B**. L2_D, **C1**. L6_D, **C2**. L6_U, **D**. L12_D. **E**. R0.5_D, **F1**. R2_D, **F2**. L2_U, **G**. R6_D, **H1**. R12_D, **H2**. R12_U. L/R0.5, 2, 6, 12: leaf/root at 0.5, 2, 6, 12 h after the onset of treatment; respectively. U, D, C: up, down, change GO terms; respectively.

To have a general view of the whole picture, we grouped and classified them into Gene Ontology terms using GoMiner (Ashburner et al. 2000; Zeeberg et al. 2003) then biological processes were further examined. From now on, mentioning a GO term means genes belonging to that GO. 10,537 rice genes in this study enriched into 284, 132 and 97 significantly changed biological processes, molecular functions and cellular components; respectively (Additional file 3: Table S3a, b, c; respectively). For the 284 biological process terms, hierarchical clustering (Figure 2) was performed with Acuity 3.1 from the converted value of GO terms (Additional file 3: Table S3d) and TreeMap view between them (Figure 3) was visualized with REVIGO (Supek et al. 2011).

Figure 2 summarizes gene expression of leaf and root in GO terms. At the earliest time point, there was only 1 significantly repressed GO in leaves while roots showed 12 indicating the earlier response of roots. It is likely because of the faster dehydration on root represented by higher fresh weight loss (Figure 1); in other words, roots are more water stressed than leaves. In the field, root also is the earlier organ under drought because plants uptake water through their roots. When soils become dry, roots sense this water deficit and produce chemical signals to transport to leaves via the xylems resulting in physiological changes in leaves (Malladi and Burns 2007; Schachtman and Goodger 2008). In this experiment, L2 started to show the rolling symptom (Additional file 1: Figure S1) indicating the decrease of cell turgor due to water loss. However, leaf tissue only gave the positive regulation at 6 h while induction was found at 2 and 12 h in root. On the other hand, repression could be found at any time point in both tissues. The Venn diagram shows that numbers of up/down regulated and changed (U/D and C) GO terms in leaf were over-represented by those in root in all three categories (Figure 2B). Thus, even though having advantages in the number of up-regulated genes (3,922 over 2,147) which were collected through the whole time range (Additional file 1: Figure S2a), in term of statistically affected GOs, leaves were out-numbered by roots. This finding suggested leaves up regulated genes in a random/scattered manner that lead to less statically significant results. Compared to that, roots seemed to behave in a more focused response. Anyway, as early as 30 min, the transcriptome of both tissues were collectively moving toward the ‘responses’ terms by changes of both up- and down-regulation. This may indicate the effort of plants to reprogram and prepare against harsh conditions as soon as 30 min (or less).

The detail definition of significant change is presented in Figure 3. At 30 minutes, no term was significantly up-regulated, only “DNA metabolic processes” was down-regulated in the leaf. However, GO terms of changes, which were calculated considering both up- and down-regulated genes were already responding toward stresses. Most significance was responses to stress and heat (Figure 3A). Unlike the responses of the leaf, root tissue showed notable down-regulations, most related to protein phosphorylation and enzyme link receptor protein signaling pathway (Figure 3E). The 27 terms of ‘Change’ in root related to recognition of stress (radiation, light, abiotic), signal transduction, and other responses. Even though no separate activation was enriched enough, general change in this time point still give off the importance of stress perceptive and signal transduction as a preparation step. After that, the changes are supposed to clearly separate between up- and down- regulation in order to have effective adaptation to stress.

Leaves at 2 h mainly repressed the expression of genes involved in photosynthetic function (photosynthesis, pigment biosynthesis, chloroplast organization) and secondary metabolism, whose products are important for plant growth and development (Figure 3B). Besides that, plant type hypersensitive response was also strongly inhibited. The data suggest that the leaves were trying to reduce processes functioning in normal condition and re-direct the whole system on responding to stress. In contrast, root tissues became more active with 41 U over 58 C and 68 D terms. The response to stress was detailed, with many up-regulated terms (Figure 3F2), suggests the dynamic response of rice root at this time point. Surprisingly, while being repressed in leaf, photosynthesis and related processes were among enhanced processes in root. In term of individual genes, some identical genes showed the opposite expression pattern in leaf and root indicating the tissue-specific regulation of these photosynthetic genes during dehydration stress. Others were biosynthesis of abscisic acid and carotenoid, metabolism of isopropenoid and lignin. The repression in root at 2 h (Figure 3F1) also gave more information than at 30 min. Not only protein modification, DNA replication and DNA/rRNA metabolism were strongly inhibited.

Leaf at 6 h and 12 h after the onset of stress treatment continued repressing similar process as 2 h (Figure 3C1, D). Among time range examined, only leaf_6 h showed the up regulation which were response to (heat, chemical) stimulus and lignin biosynthesis. Besides, aromatic compounds, farnesyl diphosphate, and isoprenoid biosynthesis were also active (Additional file 3: Table S3b). In the other part of the plant, down-regulation was major response in the root at 6 h with the continuation of processes from 2 h (Figure 3G). And those processes which are related to growth and development were still negatively regulated at 12 h (Figure 3H1). Induced GO terms are also similar to those at 2 h (Figure 3H2, Additional file 3: Table S3b).

Among the GO terms commonly induced in both leaves and roots (Table 1), except lignin and isoprenoid metabolic processes, others are directly related to the term of “response to stress”. Biosynthetic processes of an aromatic compound and farnesyl diphosphate were specific to leaves, while roots showed their own specific terms, which were related to more detailed stresses and the biosyntheses of thiamin, ABA, and carotenoids (Additional file 1: Table S4). In particular, the simultaneous reduction in leaf and induction in root of photosynthesis as well as secondary metabolism suggests the possibility of biological processes functioning in an opposite and tissue-specific manner. In agreement with the dominance of number of repressed over activated genes, the common repressed GO terms between leaves and roots are more abundant compared to induced ones. They are mainly involved in gene expression and negative regulation as well as some signaling pathways (Table 1).

**Table 1:** Commonly regulated GO terms in leaf and root of rice

**Up In L and R**	**Leaf**	**Root**
GO:0006950_response to stress	0.0054	0.0006
GO:0050896_response to stimulus	0	0.0006
GO:0042221_response to chemical stimulus	0	0.0049
GO:0009628_response to abiotic stimulus	0.0047	0.0009
GO:0009408_response to heat	0	0
GO:0009266_response to temperature stimulus	0	0
GO:0009808_lignin metabolic process	0.0017	0.0351
GO:0009644_response to high light intensity	0.029	0
GO:0006720_isoprenoid metabolic process	0.0358	0.0341
GO:0009719_response to endogenous stimulus	0.0361	0.0296
GO:0009725_response to hormone stimulus	0.0359	0.0122
GO:0009642_response to light intensity	0.0493	0
**Down in L and R**	**Leaf**	**Root**
GO:0006468_protein amino acid phosphorylation	0	0
GO:0048544_recognition or rejection of self pollen	0	0
GO:0009875_pollen-pistil interaction	0	0
GO:0008037_cell recognition	0	0
GO:0016310_phosphorylation	0	0
GO:0006950_response to stress	0.0003	0
GO:0044237_cellular metabolic process	0.0017	0
GO:0006259_DNA metabolic process	0.0022	0
GO:0042254_ribosome biogenesis	0.037	0
GO:0050896_response to stimulus	0	0
GO:0034470_ncRNA processing	0	0
GO:0043412_biopolymer modification	0	0
GO:0043687_post-translational protein modification	0	0
GO:0006464_protein modification process	0	0
GO:0006793_phosphorus metabolic process	0	0
GO:0006796_phosphate metabolic process	0	0
GO:0008152_metabolic process	0.0113	0
GO:0016072_rRNA metabolic process	0.0117	0
GO:0006261_DNA-dependent DNA replication	0.0022	0
GO:0006364_rRNA processing	0.013	0.0008
GO:0006952_defense response	0	0
GO:0051704_multi-organism process	0.0055	0
GO:0007166_cell surface receptor linked signal transduction	0.0054	0
GO:0008283_cell proliferation	0.0023	0.0062
GO:0007169_transmembrane receptor protein tyrosine kinase signaling pathway	0.002	0
GO:0009856_pollination	0	0
GO:0007167_enzyme linked receptor protein signaling pathway	0.0022	0
GO:0006270_DNA replication initiation	0.0378	0.0032
GO:0006396_RNA processing	0	0.021
GO:0043283_biopolymer metabolic process	0.0054	0.0176
GO:0016481_negative regulation of transcription	0.0127	0.022
GO:0010629_negative regulation of gene expression	0.0185	0.0234
GO:0009890_negative regulation of biosynthetic process	0.0185	0.0443
GO:0010558_negative regulation of macromolecule biosynthetic process	0.0277	0.0376
GO:0010605_negative regulation of macromolecule metabolic process	0.0285	0.0484

Numbers shown are minimum FDR value among the whole set of different time point’s value of each GO term.

### Leaf and root share no common activation on cellular metabolism

Cellular metabolism is the center of life. To see how the stress affects to the biochemical network of cells, we retrieved *Arabidopsis* homologs of rice genes from aracyc_dump_20051021 (http://www.Arabidopsis.org/biocyc) and calculated the changed enzymes in each pathway. Table 2 shows the number of up (U)- or down (D)-regulated enzymes in pathways that have at least two changed (C) enzymes, the total number of affected enzymes are shown in the C column if the numbers of U and D are smaller than 2. The majority of changed enzymes in rice was repressed with more affected enzymes were found in leaf. Some pathways were significantly affected in both parts; they’re involved in energy production, biosynthesis of phytohormones, amino acids, pigments and many others. Many were co-repressed in both tissues, such as carbon monoxide dehydrogenase pathway, de novo biosynthesis of purine nucleotides I, tRNA charging pathway or chlorophyll biosynthesis.

**Table 2:** Significant affected biochemical pathways under drought stress in rice

**Pathway**	**Leaf**												**Root**											
	**0.5 h**			**2 h**			**6 h**			**12 h**			**0.5 h**			**2 h**			**6 h**			**12 h**		
	**U**	**D**	**C**	**U**	**D**	**C**	**U**	**D**	**C**	**U**	**D**	**C**	**U**	**D**	**C**	**U**	**D**	**C**	**U**	**D**	**C**	**U**	**D**	**C**
Aerobic respiration		2		2										3			2			4			5	
Gluconeogenesis		2		2	2		2	2		2				2			2			2			3	
De novo BS of pyrimidine ribonucleotides				2				3			3									2			2	
Mevalonate pw			**2**				2								**2**									
Chorismate BS				2			2			2				2			2			2			2	
Tryptophan BS					2		2	2			2						2			2			2	
Photorespiration		2			3			3			3								2					
Brassinosteroid BS II									**2**			**2**				2								**2**
Cytokinins 7-N-glucoside BS						**2**			**2**			**2**	2								**2**			**2**
Carotenoid BS					2			2				**2**				3			3			3		
Starch degradation		2									2			2									2	
IAA BS I											3												2	
Ascorbate glutathione cycle			**2**		2			3			3			2							**2**		2	
Carbon monoxide dehydrogenase pw		7			6			9			9			2			2			4			6	
De novo BS of purine nucleotides I		4			4			6			8						2			7			7	
dTDP-L-rhamnose BS		2						2			2												2	
Phospholipid BS		3			2			2			2			2									2	
Sterol BS		2			2			2			2									2			2	
FormylTHF BS		2						3			3			2						2			3	
Glycine BS		2				**2**		2			2			2										**2**
Cysteine BS		2									3												2	
Glycine degradation								2			2						2			2			2	
Tetrahydrofolate BS II								2			2										**2**			**2**
Triacylglycerol degradation		2			6			6			6			3				**2**			**2**		3	
tRNA charging pw		4			3			5			5			2						2			6	
Dolichyl-diphosphooligosaccharide biosyn											2			2										
Chlorophyll BS		2			2			6			6			3			3			5			6	
Lignin BS									**2**			**2**								4			4	
GDP-D-rhamnose BS									**2**											2			3	
Choline BS III				3			2			2														
Trehalose BS				2			2			2														
Asparagine degradation I								2			2													
Calvin cycle								2				**2**												
Fatty acid elongation _ saturated											2													
Glycolipid BS						**2**						**2**												
Lysine BS					2			2			2													
Pantothenate BS					2			4			3													
Threonine degradation									**2**			**2**												
Cellulose BS																	3			3			2	
De novo BS of pyrimidine ribonucleotides																				2			2	
Fatty acid BS _ initial steps																	2			2			2	
Homoserine BS																				2			2	
Isopentenyl diphosphate BS _ mevalonate-independent																							2	
Jasmonic acid BS																					**2**			
Riboflavin and FMN and FAD BS															**2**									
Ribose degradation																								**2**

U/D: up/down-regulated, BS: biosynthesis, p/w: pathway. Total U, D and C enzymes in leaf are 75, 254, and 329; in root are 38, 193, and 231; respectively.

However, no pathway was found to be co-activated in both tissues. Chorismate biosynthesis or mevalonate pathway could be considered to be up regulated in leaves while they were downed or slightly changed; respectively, in roots. Vice versa, carotenoids biosynthesis, photorespiration, or brassinosteroid (BR) biosynthesis II, cytokinins 7-N-glucoside biosynthesis were activated in roots while repressed, or generally changed in leaves; respectively. Interestingly, gluconeogenesis had a tendency that gradually changed from repression to activation as drought treatment increased from 30 min to 12 h in leaves suggesting the positive function of glucose in progressive water loss. This pathway is stably repressed in roots.

Not only did each tissue differently regulate genes within the shared pathway, they also possessed tissue-specific reactions. Particularly, leaf showed strong induction of choline and trehalose biosyntheses when cellulose and initial step of fatty acid biosynthesis were negatively regulated only in root.

### Transcription factors are enhanced more than metabolism

TF might be main components to understand the complexity in signaling and controlling the expression of stress induced genes as suggested in many previous studies (Shinozaki and Yamaguchi–Shinozaki 2000; Agarwal et al. 2006; Wang et al. 2008; Hussain et al. 2011; Lata et al. 2011; Hu et al. 2013; Liu et al. 2013). In order to further investigate on these molecules, we calculated the number of up and down-regulated TFs using Conserved domain database (http://www.ncbi.nlm.nih.gov/Structure/cdd/cdd.shtml). Similar to Table 2, numbers of U, D and C TF are shown in Table 3. Compared to metabolism enzymes, TF were more highly activated as more genes were up regulated and widely distributed in both leaf and root. Among 26 groups that are affected by drought, 15 contained up regulated genes. Myb_DNA-binding, zf-C3HC4, and NAM were topmost strongly affected groups even though the balance between U and D genes in these groups are different between the 2 tissues. Most of groups containing U TF also had D genes, which leads to the variation in changing tendency of the whole group at different time point. Only HSF_DNA binding kept the activation with no repression through the whole time range in both leaf and root. In the other hand, WRKY, K-box, and CCT seemed to be stably enhanced in leaf and root; respectively. Among groups that are repressed in both tissues, significance is auxin response and GRAS which showed stable repression and no significant up regulation. Tissue-specific affected groups only contained D or C genes which are mainly involved in normal transcription process of the cell.

**Table 3:** Significant affected transcription factor, TF, families during drought response

	**Leaf**												**Root**											
	**0.5 h**			**2 h**			**6 h**			**12 h**			**0.5 h**			**2 h**			**6 h**			**12 h**		
	**U**	**D**	**C**	**U**	**D**	**C**	**U**	**D**	**C**	**U**	**D**	**C**	**U**	**D**	**C**	**U**	**D**	**C**	**U**	**D**	**C**	**U**	**D**	**C**
Myb_DNA-binding	**4**	3		**12**	5		**8**	3			2		**2**	8		**4**	8		**5**	7		**5**	9	
zf-C3HC4		3			2		**11**	3		**9**	2					**2**	6		**3**	8		**3**		
NAM		4		**11**				2		**13**	3			8		**5**	5		**6**	5		**6**	5	
HSF_DNA-binding	**2**			**2**			**2**			**2**			**3**			**2**			**2**			**2**		
WRKY			2	**3**			**2**			**2**				4				2		3		**2**	3	
K-box				**3**			**2**											2			2			2
CCT		2			2		**2**	3		**2**	2					**2**			**2**			**2**		
F-box		4		**3**	2		**2**			**2**				2		**2**			**2**					2
NmrA		3		**3**	3		**2**	2				2		5		**3**			**3**	2		**2**	2	
Homeobox	**2**	4		**2**	4		**2**	5			5			4		**2**	2			2			2	
AP2	**2**	2		**3**			**4**	2		**2**	2							2		2			3	
bZIP_1		2								**3**								2			2			
SRF-TF				**2**				2				2								3			3	
GRAS						2		2			2			2			3			3			2	
Auxin_resp					3			3			3			3									3	
mTERF								2			2			2										
YL1			2			2			2		2													2
AUX_IAA								2				2											2	
B3									2			2												
MFMR						2			2			2												
Sigma70_r2					3			3			3													
PAZ		2			2			2			2													
CDC50		2																						
BAF1_ABF1																					2			2
TFIIA														3			3			3			3	
E2F_TDP														2			2			2			2	

Total U, D and C TF in leaf are 171, 185, and 356; in root are 104, 212, and 316; respectively.

### Photosynthesis gene is induced in root under dehydration

The expression of photosynthesis related genes in root were lower than in leaf. However, the expression pattern is very different between two organs. Contrary to the observed repression in leaves, photosystem, PS, (especially light reaction) was induced in the root (Additional file 3: Table S3a).

Photosynthesis genes that are encoded separately in plastid and nuclear genome are responsible for PS core subunits, and light harvesting complex proteins and pigment biosynthesis; respectively (Kobayashi et al. 2013). While “GO:0046148_pigment biosynthetic process” showed no significant change in root, “GO:0009765_photosynthesis, light harvesting” was enhanced in this organ and both of them were repressed in leaf (Additional file 3: Table S3a). It is well-known that light and plastid development are both crucial for the expression of nuclear photosynthesis genes. However, Sullivan and Gray (1999) reported that plastid-derived signal is synthesized and is able to regulate nuclear photosynthetic gene expression in the absence of light in both pea and Arabidopsis. Although no biological process GO shows the activation of plastid-related biosynthesis in root, we found that 20/30 up-regulated GOs in Cellular Component category are related to chloroplast and plastid especially 10 contains “thylakoid” (Additional file 3: Table S3c) which was suggested to be very important to the development of chloroplast (Kobayashi et al. 2013).

Related to 2 groups of nuclear encoded genes, pigment biosynthetic process was down-regulated in leaf but didn’t show significant change in root; while light harvesting was inhibited in leaf and activated in root (Additional file 3: Table S3a). Looking into Molecular Function GOs, we found “GO:0016168_chlorophyll a/b binding” protein (Additional file 3: Table S3b) was significantly up-regulated in root while repressed in leaf. Among 18 members of this gene family in rice (Umate 2010), 6 genes were up-regulated in all examined time points and their expression pattern was certified by the semiquantitative (sq) RT-PCR as following.

**Figure 4:**
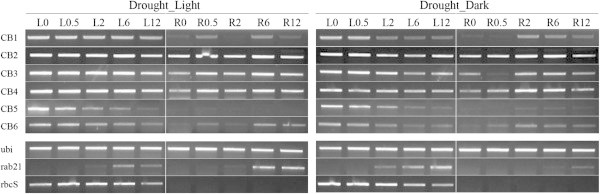
**Expression patterns of chlorophyll binding (CB) genes under dehydration treatment.** The experiment was carried out in light (left) or dark (right) conditions. L/R0, 0.5, 2, 6, 12: leaf/root at 0, 0.5, 2, 6, 12 h after the stress onset.

Because our experiment treated the whole seedling in a chamber under continuous light, there is a possibility that photosynthesis related genes were induced because roots were exposed to light even though they were covered by a layer of tissue and aluminum foil. To test whether their activation is induced by dehydration or light, we repeated the experiment and also conducted a similar acute dehydration treatment as using in the microarray experiment but under dark condition. Figure 4 shows the expression patterns of the above 6 genes in two dehydration experiments (with and without light). Their expression in the dark was lower than in the light; however, clear induction was still observed. This primary result may lead to a very interesting study on the function of photosystem genes in root during water loss stress.

### Energy condition in leaf during dehydration

**Figure 5:**
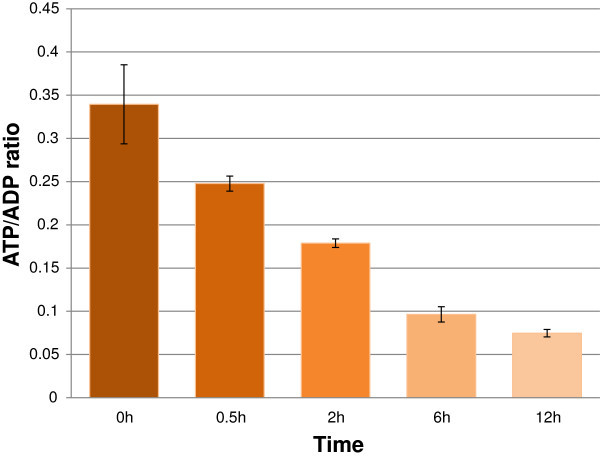
**ATP/ADP ratio of rice shoot during dehydration.** Value = mean ± SD (n = 3).

**Figure 6:**
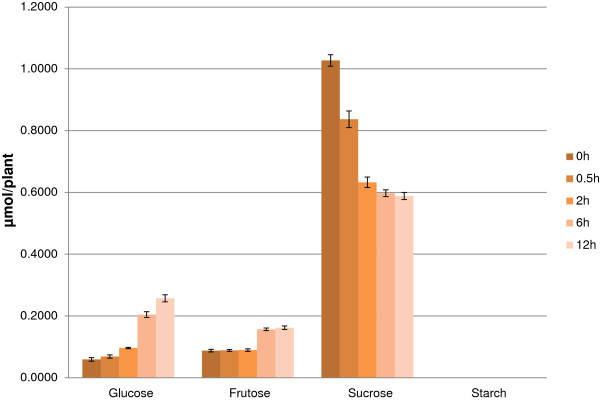
**Starch and ethanol-soluble sugars content in rice shoot during acute dehydration.** Value = mean ± SD (n = 3), y value axis unit is μmol/plant.

The repression found on many growth and development processes, which require high energy level, might impose the lower level of energy compared to normal. Indeed, Figure 5 shows the rapid decrease of ATP/ADP ratio of rice during stress duration indicates the lack of energy in this situation. Because of the inhibition of photosynthesis during stress as reported above and in many previous studies (Degenkolbe et al. 2009, Shu et al. 2011), the plant must mobile energy from storage resources such as carbohydrates, fatty acids and proteins. Among the enzymes that catalyze the breakage of complex substrates into simpler molecules to produce energy, hydrolase was found to be enhanced in leaf during dehydration stress (Additional file 3: Table S3b). Especially, the up-regulation of “GO:0004553_hydrolase activity, hydrolyzing O-glycosyl compounds” suggested the important of carbohydrate-derived energy. As a carbohydrate, starch is intermediate-term storage of energy in plant. However, the starch content of 14d rice seedling is too small for any changes to detect (Figure 6). Besides, starch degradation was down regulated as in pathway analysis (Table 2). Thus, starch doesn’t seem to be an important energy resource in this case, which used 14d rice seedlings. Besides starch, sugars are short-term energy storage, among the 3 ethanol-soluble sugars examined; sucrose has highest content and shows the decrease pattern while glucose and fructose are increased by treated time. This suggests sucrose as an important energy resource of rice seedling during dehydration.

## Discussion

### Response of rice seedling to acute dehydration

Rice appeared to respond to stress mainly by down-regulating many biological processes. Additionally, rice also showed a predominance of root over leaf in Under, Over, and Change GO terms. Although the number of up-regulated genes in the leaf was higher than in the root (3,922 over 2,147), the induction was obtained at only one time point, 6 h with 16 “biological process” type GO terms. Alternatively, induction in roots enriched in more GO terms (43), and wider time range (2 and 12 h). This observation suggests the large difference in the response of two tissues to water deficiency.

As other plants under stress, rice seedling also uses popular mechanism to protect itself from damage. One of the earliest responses is the change in protein phosphorylation status, so phosphorylation-related GOs were easily found in both tissues at various time points. Besides, there were gene’s products that directly protect against environmental stresses, including proteins that likely function in stress tolerance, and those that regulate gene expression (transcription factors) and signal transduction (phytohormones) in the stress response. The first step after plant perceives stress is signal transduction, which can be done by plant hormones. ABA is believed to play a major role in plant responses to stresses that there are ABA-dependent and ABA-independent pathways. Cytokinin and BR may be component of the latter pathways. Cytokinin has been suggested to increase tolerance to mild stress and accelerate a plant’s recovery during drought by applying exogenous cytokinin. In addition, there is a potential role for overexpression of cytokinin in root during the plant’s drought response (Novakova et al. 2007). The exogenous application of BR also improved drought tolerance in rice (Farooq et al. 2009).

Downstream of signal transduction is the expression of genes. One group is TF_regulatory proteins, another one is functional proteins. Among TFs that were changed by drought, Myb_DNA_binding, zf-C3HC4, and NAM were most strongly affected. Various Myb-type TFs have been proved to be induced and function in drought response (Bartels and Souer 2003). MYB in a combination with MYC works as a system in ABA-dependent pathway (Agarwal et al. 2006). C3HC4 zf is RING-type zinc finger, some of whose have E3 ubiquitin-protein ligase activity determining the substrates specificity for ubiquitination, an important step in protein modification. C3H zinc finger family has been reported to be regulated by abiotic or biotic stresses (Wang et al. 2008). NAC (NAM, ATAF1/2, CUC2) is a new plant specific transcription factor and play diverse roles in plant development and stress responses. Recently, its involvement in salinity tolerance was reported (Agarwal et al. 2006).

The group of functional molucules includes trehalose, choline, carotenoid, and thiamin. Trehalose, a disaccharide, is accumulated in plants, fungi, and invertebrates and functions as a compatible solute to protect their biological structure under severe stress conditions (Garg et al. 2002). Choline is a precursor for the formation of glycine betaine, which can act as osmoprotectant in plant and confers tolerance to salinity, drought, and other environmental stresses (Kishitani et al. 2000; Shirasawa et al. 2006). In another way, carotenoid is a non-enzymatic antioxidants that function in maintaining the integrity of photosynthetic membranes (Munné-Bosch and Penuelas 2004), protecting them from the oxidative stress which is the result of an imbalance between antioxidant defenses and activated oxygen species during drought. Vitamin B1 (thiamine) is also suggested to participate in the processes underlying plant adaptations to certain types of abiotic and biotic stress, mainly oxidative stress (Rapala-Kozik et al. 2012). The resulted oxidative stress is also likely to be the reason of HSF_DNA-binding TF induction because HSFs might be involved not only in heat shock protein synthesis but also in oxidative stress regulation of antioxidant gene expression (Wang et al. 2004).

Coordinate with activation of protective mechanism, to save energy and focus on drought response, genes involved in growth and development should be repressed. This can explain for the down-regulation of many synthesis processes such as photosynthesis, cell wall, nucleotide and ribonucleotide, lipid and protein synthesis, as well as TFs that are mainly related to development like GRAS and Auxin-reponse factors.

### Tissue-specific production and possible transportation mechanism of functional products

Although leaf and root share many common terms related to responses to different kind of stresses (Table 2), cellular metabolism and TF synthesis seemed to be highly tissue-specific. Among two groups of protective molecules induced in rice under drought, osmoprotectant was found only in leaves while antioxidant was dominant in roots. Moreover, if we consider a TF group is activated or repressed at one time point when the number of U TF is more or less than that of D TF at the same time, respectively; the up regulation of TFs was mostly observed in leaves and repression was the main tendency in roots. In contrast, hormone biosyntheses were only induced in roots.

Interestingly, among pathways and GO terms that are more induced in leaves over roots, except choline and trehalose that directly function in drought, other processes’ products are precursors of many important cellular biosynthesis. The mevalonate pathway leads to sterol biosynthesis, ergosterol biosynthesis, and dolichols *via* the formation of farnesyl diphosphate (FPP). In plants, the mevalonate pathway is also a source of isoprene units for the biosynthesis of a variety of terpenoids (cytokinins, brassinosteroids, sesquiterpenes, polyprenoids or carotenoid, a tetraterpenoid) (Bochar et al. 1999). The chorismate biosynthetic pathway links the metabolism of carbohydrates to the biosynthesis of proteinogenic aromatic amino acids (phenylalanine, tyrosine, and tryptophan). This pathway is also thought to modulate the carbon flux from ‘primary metabolism’ to ‘secondary (specialized) metabolism’ because chorismate represents the end product of the pathway, which gives rise to a number of specialized metabolites such as quinones, phenylpropanoids, and indoles (e.g., IAA biosynthesis I) (Weaver et al. 1997). Easily to be seen, most of protective molecules produced in roots need precursor unit from those two pathways, which are induced in leaves. Thus, there is necessary presence of (a) transportation mechanism(s) that quickly and effectively transport(s) those molecules from the source (leaves) to the sink (roots). Similarly, TFs and hormones which are produced in this site of seedling also need to be transported to the other site to function.

### Photosynthesis and chlorophyll a/b binding proteins in root during acute dehydration stress

A significant change during drought response was the repression of PS gene expression in the leaf, and this finding supports previous data on the response of rice during drought (Degenkolbe et al. 2009, Shu et al. 2011). As mentioned above, oxidative stress occurs during drought. Because of the over-reduction of reaction centers and the increased production of active oxygen species in chloroplasts, PS (especially PSII) appears to be in excess energy status and this occurrence can be harmful, if not safely dissipated (Loggini et al. 1999). In this study, we found that PS (especially light reaction) was induced in the root, which was not clear when the expression of root was directly compared to that of leaf.

Plant roots usually grow underground as heterotrophic organs and are considered as non-photosynthetic organs. Roots may become green when exposed to light or when they develop as adventitious organs. In roots of the epiphytic Orchidaceae and in the aerial roots of mangroves, photosynthesis by this organ does, in fact, contribute to the carbon economy of the whole plant (Flores et al. 1993). Mature rice also produced aerial roots called adventitious prop roots. Although 14d seedling hasn’t produced adventitious prop roots, the exposure of the whole plant into air in this experiment made roots similar to aerial. In the field condition, when water is withdrawn, there’s possibility that roots are exposed to the air and in this case, this part of root can activate photosynthesis to compensate for the reduction of this activity in leaf.

Among photosynthesis genes, “GO:0016168_chlorophyll a/b binding protein” was induced in root and 6 genes were consistently up-regulated in all examined time points. The general function of the chlorophyll a/b binding family is to absorb light and transfer its energy to the photochemical reaction core. By binding to free chlorophyll molecules, they can prevent the formation of free radicals and/or acting as sinks for excitation energy. Therefore, these genes were also proposed to have a protective role within the thylakoids during light stress (Andersson et al. 2003). The pathway analysis showed that chlorophyll biosynthesis was strongly down-regulated (Table 2), suggesting an interesting theory that those gene products that actually do function in drought response might not function by binding to chlorophyll a/b, but by an alternate mechanism, a chlorophyll-independent manner.

**Figure 7:**
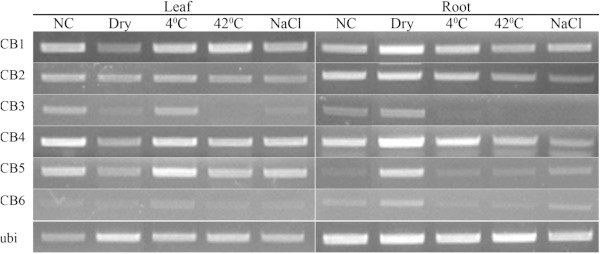
**Expression patterns of chlorophyll binding (CB) genes in rice seedlings under different abiotic stresses.** The amplification cycles were 25 and 35 for leaf and root sample; respectively. NC: non-stressed control, Dry: air-dry, 4°C: cold stress, 42°C: heat stress, NaCl: salt stress.

Generally, light-harvesting chlorophyll a/b binding, LHCB, gene expression is regulated by multiple environmental and development cues including mainly light, oxidative stress, chloroplast retrograde signal, circadian clock, and the phytohormone abscisic acid (Xu et al. 2012). In this research, they seemed to be dehydration stress specific (Figure 7) which solidified their necessary role in this kind of stress.

### Energy resources during acute dehydration

In plant, the most popular carbohydrates energy storage is starch which is biosynthesized by photosynthesis. Beside starch, sugars are also good storages of energy even though shorter-term. In stress condition, usually the abundant starch is degraded to supply energy for plants and soluble sugars can be accumulated to act as osmoprotectants (Mohammadkhani and Heidari 2008). However, 14d seedling leaves used in this study has such a low content of starch that the applied method can neither detect correctly the concentration nor distinguish the changes. In this case, sucrose has highest concentration and seems to be an important resource as its level decreases while glucose and fructose increase by time. The reason of using sucrose as main energy resource lines in the development stage of rice sample. As reported by Wopereis et al. 2009, the period of about 2 weeks after germination, young seedling essentially feeds on the food reserve in the endosperm. Thus, the component of energy resource in 14d seedling would highly be the result of transportation activity from endosperm to seedling through the scutellum (Aoki et al. 2006; Scofield et al. 2007). Sucrose is the major soluble sugar component in dry seed and generally agreed to be the major form of sugar translocated in plant. It decreases after several days of seed germination then increases again together with the accumulation of glucose, fructose and maltose (Murata et al. 1968) because during germination, starch in endosperm is broken down to produce glucose and low amounts of maltose and sucrose. The resulted glucose and maltose move to the scutellum and is converted to sucrose and then transported to shoot and root (Scofield et al. 2007). Besides, there is also the transportation of sucrose produced from lipids degradation in aleurone layer into endosperm, then to scutellum (Aoki et al. 2006). Thus, the sucrose transported to growing tissues contains both original sucrose and inverted glucose. In shoot, sucrose will be degraded to produce glucose, which is the favorite form of energy source in living cell, and fructose. Besides, the slowly activation of gluconeogenesis in leaf, as in pathway analysis, also increases glucose concentration. The total glucose is partially used to produce trehalose which is the combination of a UDP-glucose and a glucose-6-phosphate. Another function of glucose is likely to act as an osmoprotectant itself, and fructose may also involve in this mechanism.

## Conclusions

Water deficiency is one of the most serious worldwide problems for agriculture. Recently, it has become more serious and outspread, which urgently requires the production of drought-tolerant plants. Microarray experiments using mRNA from air-dried leaves and roots of rice were performed in an attempt to study genes involved in dehydration response. Leaf and root tissues shared some common gene expression during stress, with the purpose of enhancing protective systems. However, these two tissues appeared to act differently in response to the different level of dehydration they experience. Leaves induced more genes than root but those genes scattered in many processes, most significantly were productions of osmoprotectant. Roots up-regulated fewer genes and focused on inducing antioxidant and enhancing photosynthesis related genes. Besides, the two tissues can affect each other via the signaling and transportation system. Our analysis suggested an important role for the chlorophyll a/b binding proteins in drought. Further analysis would reveal more candidates for stress-inducible genes. Especially, genes identified from transgenic rice may provide more information about the affect of drought response mechanisms on gene functions, *cis*-acting elements and downstream genes in cascades.

## Methods

### Plant growth and stress treatment design

Non-transgenic rice seeds (*Oryza sativa* L. *japonica* cultivar Nakdong) were geminated on MS0 (Murashige and Skoog) agar media (0.25% phytagel) and incubated in growth chamber (28°C, 2 days in dark followed by 1 day in light). Seedlings were then transferred into soil and grown in greenhouse (16-h-light/ 8-h-dark cycle) for 11 days. They were then removed from soil, kept in fresh water for 1 day to avoid the transient gene expression of any responses to possible root injury, and then subjected to air-dry in a growth chamber with temperature of 32°C, humidity of 50% and continuous fluorescent light of 150 μmol.m^-2^.s^-1^. Although 32°C is not optimum temperature for rice development, it was chosen as the temperature of green house during the experiment time varied from 24°C (lowest at night) to 35°C (highest at noon). During the treatment, roots were covered by a of tissue and then a layer of aluminum foil to mimick the root natural condition which was deep under the soil in dark with little air contact. After 0, 0.5, 2, 6, 12 h, leaves and roots were collected separately. A similar sampling method was used for other experiments in this study.

For other stresses, seedlings at the same stage were kept in water and incubated at 4°C and 42°C to create cold and heat stress; respectively. In salt stress, water was added NaCl to a final concentration 400 mM and maintained in the same chamber of air-dried and control plants, which were kept in water. Leaves and roots were collected after 6 h of the treatment.

### Rice 300 k 3′-tiling microarray

Total RNAs were extracted using TriReagent (Molecular Research Center, Inc.). Using these RNAs, expression profiling was then conducted with the rice 3′tiling microarray analysis. Two biological replicate were done. Expression profiling was conducted using the Rice 3′-Tiling microarray manufactured by NimbleGen (http://www.nimblegen.com), which contains 27,448 genes deposited at the International Rice Genome Sequencing Project Rice Annotation Project 1 database (http://rapdb.dna.affrc.go.jp). Further information on this microarray, including statistical analysis, can be found at http://www.ggbio.com (GreenGene Biotech, South Korea).

Direct comparison of leaf and root data could be misleading because each tissue would undergo distinct response mechanisms. To overcome this, leaf data were compared to the RNA obtained at initial stage of leaf and designated Leaf 0 (L0). Similarly, root data were compared to the sample of Root 0 (R0). The comparison of leaf and root was performed using L0 and R0. Multiple analysis was performed with LIMMA package in R computing environment (Smyth 2004). The package adopts the linear modeling approach implemented by lmFit and the empirical Bayesian statistics implemented by eBayes. Genes of which adjusted-P-value or false discovery below 0.05 were collected and further selected for those gene expressions were higher than 1 or less than -1 at least at one stage compared to that at stage 1. Multivariate statistical tests such as clustering, principal component analysis, and multidimensional scaling were performed with Acuity 3.1 (Axon Instruments). Hierarchical clustering was performed with similarity metrics based on squared Euclidean correlation and average linkage clustering was used to calculate the distance of genes.

The data discussed in this publication have been deposited in NCBI’s Gene Expression Omnibus (Edgar et al. 2002) and are accessible through GEO series accession number GSE38130 (http://www.ncbi.nlm.nih.gov/geo/query/acc.cgi?acc=GSE38130).

### GoMiner analysis

GO analysis was performed with GoMiner (Ashburner et al. 2000; Zeeberg et al. 2003). Those genes selected as described above were marked as 1 and -1 according to their expression modes; 2 folds increase and decrease; respectively. To find a tentative counterpart of rice gene in *Arabidopsis* genome, Blastp analysis was performed for the two species, and the genes with Score 100 or higher were considered to be the tentative counterparts. The Rice 300 k 3′-tiling Microarray contains 27,448 genes and 18,270 which are matched with *Arabidopsis* genes with score 100 and up were used as total gene set in GoMiner. First, GoMiner categorizes each gene according to their GO terms and mode of gene expressions. Modes of expression are denoted as Under, Over, and Change to present down-, up-regulated genes, and both of these ones; respectively. It calculated P-values with the one-sided Fisher exact test for the number of categorized GO terms in the total. False discovery rate (FDR) values were obtained from 100 randomizations. GO terms of which FDR are less than 0.05 at least at one time point are collected. To present GO terms in hierarchical clusters, firstly, unique GO terms are selected from GO terms. The FDR values ranging less than 0.05 to 0 scaled to 0.5 to 5. Other non-significant FDR values are manually transformed to 0. For graphical presentation, the FDR values are multiplied with 1 (red) and -1 (green) according to up- and down- regulations; respectively. Hiearchical clustering is performed with Acuity 3.1. To reduce the redundant and visualize GO terms in interactive graphs, web-based program REVIGO (Supek et al. 2011) are used with C value of 0.7, whole UniProt as database size and SimRel as semantic similarity measure. GO list of each time point mode is loaded separately and collected graphs are then edited by normal image processing program.

### Pathway analysis

Rice genes were matched to *Arabidopsis* as described above and related pathways were retrieved from aracyc_dump_20051021 (http://www.Arabidopsis.org/biocyc).

### Transcription factor analysis

Domains in rice genes are identified by rpsblast using Conserved domain database (http://www.ncbi.nlm.nih.gov/Structure/cdd/). Transcription factors are categorized based on Pfam (http://pfam.sanger.ac.uk/).

### Semi-quantitative (sq) RT-PCR analysis

RNAs were prepared with the same method used for 3′-tiling microarray. For the first strand cDNA synthesis, 5 μg of total RNA was reverse-transcribed using Super Script III First Strand kit (Invitrogen) according to manufacturer’s manual. cDNA mixture of rice was then 2-time diluted. Gene specific primers (Additional file 1: Table S5) were designed using the Primer Designer 4 software (Sci-ed. Software, NC). PCR was performed in a 20-μl solution containing a 1-μl rice cDNA aliquot, 0.25 pM gene-specific primers, 10 μl of 2× PCR master mix (Intron biotechnology, Inc.). The reaction included an initial 2-min denaturation at 94°C, followed by 20 to 35 cycles of PCR (95°C for 30 sec, 55°C for 30 sec, 68°C for 30 sec), and a final 10 min at 68°C. Afterward, 10 μl of the reaction mixture was separated on a 2% agarose gel. To obtain the reproductibility of RT-PCR, the experiment was repeated 2 times with 2 independent biological replicated samples.

### Starch and soluble sugars measurement

Control and stressed leaf blades were harvested, weighed and kept in deep freezer (-80°C) until use. Number of plants was recorded to calculate fresh weight of leaf blade per plant, coded as A. Approximately 100 mg which is then divided for A to get the number of plants (B) in that amount of each sample was used in extraction. Ethanol extracts were performed according to Lee et al. (2008). Sucrose, glucose, fructose and starch were measured using NAD(P)H-coupled enzymatic methods with Cary 300 Bio UV-visible spectrophotometer (Agilent Technologies, USA). The measured metabolite contents were normalized to each plant by dividing the results for B. Final result is average of 3 independent samples and the experiment was repeated twice.

Sugar content was scored as μmol/plant instead of μmol/g FW because FW of leaf decreases by stress treated time. Even without real increase in solute content, its level is eventually higher because of the decrease in leaf FW. Leaf FW could be used if it’s measured before dehydration treatment but that seemed impossible because intact plant was used to avoid any injuries or other shock effect. Considering plants are biological identical, concentration of sugars is normalized by number of plants.

### ATP/ADP ratio determination

Samples were prepared as in starch and sugar measurement. To determine the ATP/ADP ratio, adenylate nucleotides were firstly extracted using proteinase K method (Napolitano and Shain 2005) with minor change. Approximately 10 mg of samples was incubated with 30.9 μl of 20 mg/ml proteinase K (Invitrogen, AM 2546) in 340 μl 50 mM HEPES. The reaction was placed at 50°C for 20 min, then inactivated by placing at 80°C for 5 min and stored on ice. The supernatant was then used as triplicate to determine ADP/ATP ratio according to protocol provided by manufacturer of EnzyLight kit (BioAssay Systems, ELDT-100). Luminescence signal was measured by Glomax Luminometer (Promega, USA). The experiment was also repeated twice with 3 technical replicas.

## Abbreviations

TF: Transcription factor; ABA: Acid abscisic; PS: Photosystem; LEA: Late abundant protein; P/S/RFW: Plant/shoot/root fresh weight; UDC: Up down change; GO: Gene ontology; FDR: False discovery rate; sqRT-PCR: Semi-quantitative reverse transcriptase polymerase chain reaction.

## Competing interests

The authors declare that they have no competing interests.

## Authors’ contributions

BHN and YKK proposed the project idea. YKK did the microarray analyses. MTPT carried out the experiment and wrote the manuscript. DJH measured ATP/ADP ratio. JSJ measured the quantities of sugars in rice leaf and root. All authors read and approved the final manuscript.

## Supplementary Material

Additional file 1: Table S1 Standard Evaluation System for Rice, IRRI. Table S4: Tissue-specific up-regulated GO terms in leaf and root of rice. Numbers shown are FDR value of GO term. Table S5: Gene specific primers used for semi-quantitative RT-PCR. CB1-6: chlorophyll a/b binding protein 1–6. rab21, rbcS: stress marker, ubi: constitutive marker. Figure S1: Seedling appearance at 0 h, 30 min and 2, 6, 12 h of dehydration (from left to right). The smaller frames in the right of each panel are the magnificent of rectangulars marked in leaf and root. Figure S2: Confirmation of strongly induced genes in microarray result (a) by sq RT-PCR (b). Leaf and root and 0 h (L0 and R0, respectively) were used as non-stressed control samples. Stress treated plants (*O. sativa japonica* cult. *Nakdong*) were air dried from 30 min to 2, 6, and 12 h. ubi was used as constitutive marker. In (a), error bar = standard deviation of intensities (n = 2). Figure S3. Clustering of significant changed genes in acute dehydration of rice. 10,537 2-fold up/down regulated genes were hierarchical clustered (a) and the over-lapping parts (b) between leaf and root were examined. Click here for file

Additional file 2: Table S2 Genes that are 2 fold up or down regulated in leaf or root. Click here for file

Additional file 3: Table S3 List of are significantly affected GO terms related to Biological Process (a), Molecular Function (b), Cellular Component (c), and converted value of 284 Biological Processe terms (d). Click here for file
