# Supplementary material for: Transcriptome analysis of leaf and root of rice seedling to acute dehydration
Source: Rice (N Y). 2013 Dec 16;6(1):38. doi: 10.1186/1939-8433-6-38 (PMC3878681; doi:10.1186/1939-8433-6-38)
Supplement: Additional file 1: Table S1 — Standard Evaluation System for Rice, IRRI. Table S4: Tissue-specific up-regulated GO terms in leaf and root of rice. Numbers shown are FDR value of GO term. Table S5: Gene specific primers used for semi-quantitative RT-PCR. CB1-6: chlorophyll a/b binding protein 1–6. rab21, rbcS: stress marker, ubi: constitutive marker. Figure S1: Seedling appearance at 0 h, 30 min and 2, 6, 12 h of dehydration (from left to right). The smaller frames in the right of each panel are the magnificent of rectangulars marked in leaf and root. Figure S2: Confirmation of strongly induced genes in microarray result (a) by sq RT-PCR (b). Leaf and root and 0 h (L0 and R0, respectively) were used as non-stressed control samples. Stress treated plants (O. sativa japonica cult. Nakdong) were air dried from 30 min to 2, 6, and 12 h. ubi was used as constitutive marker. In (a), error bar = standard deviation of intensities (n = 2). Figure S3. Clustering of significant changed genes in acute dehydration of rice. 10,537 2-fold up/down regulated genes were hierarchical clustered (a) and the over-lapping parts (b) between leaf and root were examined. [file 1939-8433-6-38-S1.docx]

| **Scale** | **Leaf rolling at vegetative stage** |
| --- | --- |
| 0 | Leaves healthy |
| 1 | Leaves start to fold (shallow) |
| 3 | Leaves folding (deep V-shape) |
| 5 | Leaves fully cupped (U-shape) |
| 7 | Leaf margins touching (0-shape) |
| 9 | Leaves tightly rolled (V-shape) |

**Table S1**. Standard Evaluation System for Rice, IRRI.

| **Leaf_U** | **6h** |  |  |
| --- | --- | --- | --- |
| GO:0019438_aromatic compound biosynthetic process | 0.005 |  |  |
| GO:0009611_response to wounding | 0.0265 |  |  |
| GO:0045337_farnesyl diphosphate biosynthetic process | 0.0496 |  |  |
| **Root_U** | **2h** | | **12h** |
| GO:0015979_photosynthesis | 0.0014 | | 0 |
| GO:0019684_photosynthesis, light reaction | 0.0017 | | 0 |
| GO:0006091_generation of precursor metabolites and energy | 0.0015 | | 0.0012 |
| GO:0042542_response to hydrogen peroxide | 0.0017 | | 0.0012 |
| GO:0009767_photosynthetic electron transport chain | 0.0008 | | 0.0022 |
| GO:0015976_carbon utilization | 0.0047 | | 0.0071 |
| GO:0009768_photosynthesis, light harvesting in photosystem I |  | | 0.0147 |
| GO:0009765_photosynthesis, light harvesting | 0.0062 | | 0.0164 |
| GO:0009414_response to water deprivation | 0.0043 | | 0.0406 |
| GO:0015977_carbon utilization by fixation of carbon dioxide | 0.0198 | | 0.0447 |
| GO:0009228_thiamin biosynthetic process |  | | 0.0472 |
| GO:0022900_electron transport chain | 0.0133 | | 0.0478 |
| GO:0006470_protein amino acid dephosphorylation | 0.0456 | |  |
| GO:0006721_terpenoid metabolic process | 0.029 | |  |
| GO:0006970_response to osmotic stress | 0.0094 | |  |
| GO:0006979_response to oxidative stress | 0.0235 | |  |
| GO:0009415_response to water | 0.0046 | |  |
| GO:0009416_response to light stimulus | 0.0376 | |  |
| GO:0009651_response to salt stress | 0.0025 | |  |
| GO:0009688_abscisic acid biosynthetic process | 0.0261 | |  |
| GO:0009737_response to abscisic acid stimulus | 0.0014 | |  |
| GO:0009773_photosynthetic electron transport in photosystem I | 0.0238 | |  |
| GO:0010039_response to iron ion | 0.0432 | |  |
| GO:0016108_tetraterpenoid metabolic process | 0.0358 | |  |
| GO:0016109_tetraterpenoid biosynthetic process | 0.0128 | |  |
| GO:0016116_carotenoid metabolic process | 0.0358 | |  |
| GO:0016117_carotenoid biosynthetic process | 0.0128 | |  |
| GO:0019748_secondary metabolic process | 0.0352 | |  |
| GO:0030001_metal ion transport | 0.0438 | |  |
| GO:0042445_hormone metabolic process | 0.0205 | |  |
| GO:0043289_apocarotenoid biosynthetic process | 0.0261 | |  |

**Table S4.** Tissue-specific up-regulated GO terms in leaf and root of rice. Numbers shown are FDR value of GO term.

| **Name** | **Forward primer** | **Reverse primer** |
| --- | --- | --- |
| ADF (Os03g0820500)  AWPM (AK102039)  rab21 (AK121952)  dip1(AK070197)  rbcS (AK121444)  ubi(Os06g0681400)  CB1 (AK060904)  CB2 (AK119176)  CB3 (AK066762)  CB4 (AK060222)  CB5 (AK119534)  CB6 (AK106085) | GCCGTGTACGACCACGACTT  CTCTTCTCCGGCAACCACGC  CGTCTACCGTGAGAACCACA  GAAGCCGGAAGACGCAACTG  ACCACCGACACCGGCGAGAA  ATGGAGCTGCTGCTGTTCTA  CGTGAAGGCTATGGGTGGTT  CAACCTCTTCGCCCATCTCG  AGATGGTGCCAACCAACGTA  AGCACAACGTGACGCAGAAG  TGCAGCTGGCGGAGATCAAG  AACATCCTCACCAGCCTCAA | TTAGGAGGTGTGGTCCTTGAG  AGCTCGATCACACCGAGATA  GTAGGCGATGAAGCTGATGA  CAACCATGGCCTGGTCTCAC  TTGCGCTCGGCTAGCTCATC  TTCTTCCATGCTGCTCTACC  GGACGGATTCACAAGAGAGCA  CACCACCATTCACAGCCTAC  ATGCGGCCTGCGGCTTACAT  ACATCTTCCATCGCCCAATC  CTCGCTCGTCATCACAAAAC  ACGCACCATCGATCGTACAA |
|  |  |  |

**Table S5.** Gene specific primers used for semi-quantitative RT-PCR. CB1-6: chlorophyll a/b binding protein 1-6. rab21, dip1, rbcS: stress marker, ubi: constitutive marker.


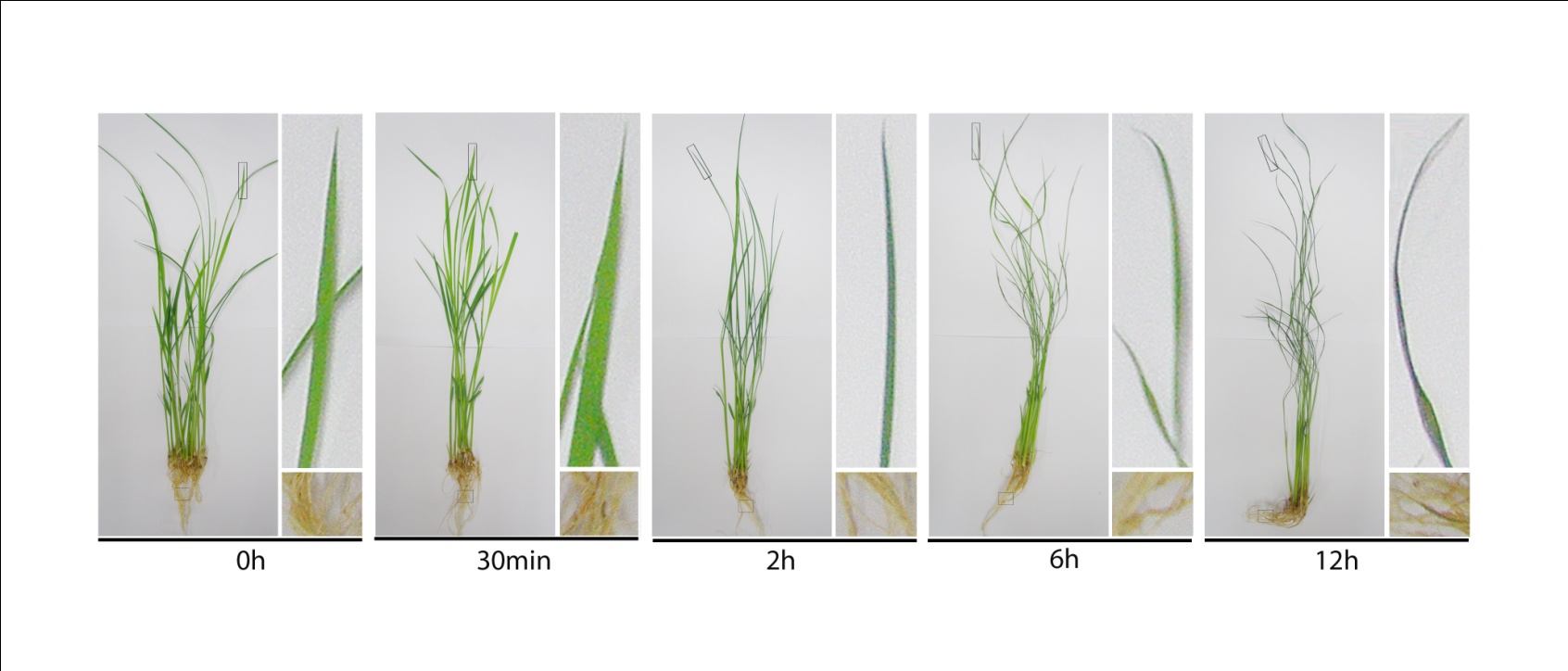


**Figure S1.** Seedling appearance during acute dehydration

Seedlings after 0h, 30 min and 2, 6, 12h of air dry (from left to right). The smaller frames in the right of each panel are the magnificent of rectangulars marked in leaf and root.

**Figure S2**. Confirmation of strongly induced genes in microarray result **(a)** by sq RT-PCR **(b)**. Leaf and root and 0h (L0 and R0, respectively) were used as non-stressed control samples. Stress treated plants (*O. sativa japonica* cult. *Nakdong*) were air dried from 30 min to 2, 6, and 12 h. ubi was used as constitutive marker. In (a), error bar = standard deviation of intensities (n=2).

**Figure S3**. Clustering of significant changed genes in acute dehydration of rice. 10,537 2-fold up/down regulated genes were hierarchical clustered (a) and the over-lapping parts (b) between leaf and root were examined.
